# Bakuchiol Is a Phenolic Isoprenoid with Novel Enantiomer-selective Anti-influenza A Virus Activity Involving Nrf2 Activation*[Fn FN2]

**DOI:** 10.1074/jbc.M115.669465

**Published:** 2015-10-07

**Authors:** Masaki Shoji, Yumie Arakaki, Tomoyuki Esumi, Shuntaro Kohnomi, Chihiro Yamamoto, Yutaka Suzuki, Etsuhisa Takahashi, Shiro Konishi, Hiroshi Kido, Takashi Kuzuhara

**Affiliations:** From the ‡ Laboratory of Biochemistry, Faculty of Pharmaceutical Sciences, and; the § Institute of Pharmacognosy, Faculty of Pharmaceutical Sciences, Tokushima Bunri University, Tokushima 770-8514, Japan,; the ¶ Department of Neurophysiology, Kagawa School of Pharmaceutical Sciences, Tokushima Bunri University, Kagawa 769-2193, Japan,; the ‖ Graduate School of Frontier Sciences, University of Tokyo, Kashiwa, Chiba 277-8568, Japan, and; the ** Division of Enzyme Chemistry, Institute for Enzyme Research, Tokushima University, Tokushima 770-8503, Japan

**Keywords:** antiviral agent, infectious disease, influenza, influenza virus, nuclear factor 2 (erythroid-derived 2-like factor) (NFE2L2) (Nrf2), virus, bakuchiol, enantiomer-selective

## Abstract

Influenza represents a substantial threat to human health and requires novel therapeutic approaches. Bakuchiol is a phenolic isoprenoid compound present in Babchi (*Psoralea corylifolia* L.) seeds. We examined the anti-influenza viral activity of synthetic bakuchiol using Madin-Darby canine kidney cells. We found that the naturally occurring form, (+)-(*S*)-bakuchiol, and its enantiomer, (−)-(*R*)-bakuchiol, inhibited influenza A viral infection and growth and reduced the expression of viral mRNAs and proteins in these cells. Furthermore, these compounds markedly reduced the mRNA expression of the host cell influenza A virus-induced immune response genes, interferon-β and myxovirus-resistant protein 1. Interestingly, (+)-(*S*)-bakuchiol had greater efficacy than (−)-(*R*)-bakuchiol, indicating that chirality influenced anti-influenza virus activity. *In vitro* studies indicated that bakuchiol did not strongly inhibit the activities of influenza surface proteins or the M2 ion channel, expressed in Chinese hamster ovary cells. Analysis of luciferase reporter assay data unexpectedly indicated that bakuchiol may induce some host cell factor(s) that inhibited firefly and *Renilla* luciferases. Next generation sequencing and KeyMolnet analysis of influenza A virus-infected and non-infected cells exposed to bakuchiol revealed activation of transcriptional regulation by nuclear factor erythroid 2-related factor (Nrf), and an Nrf2 reporter assay showed that (+)-(*S*)-bakuchiol activated Nrf2. Additionally, (+)-(*S*)-bakuchiol up-regulated the mRNA levels of two Nrf2-induced genes, NAD(P)H quinone oxidoreductase 1 and glutathione *S*-transferase A3. These findings demonstrated that bakuchiol had enantiomer-selective anti-influenza viral activity involving a novel effect on the host cell oxidative stress response.

## Teaser

**Background:** Novel therapeutic approaches against influenza are required. Bakuchiol is a phenolic isoprenoid found in Babchi seeds.

**Results:** Bakuchiol enantiomer-selectively inhibited influenza A viral infection and growth and activated the Nrf2 pathway.

**Conclusion:** Bakuchiol showed novel enantiomer-selective anti-influenza viral activity.

**Significance:** The study of bakuchiol will contribute to the development of novel approaches to influenza therapy.

## Introduction

**FIGURE 1.**
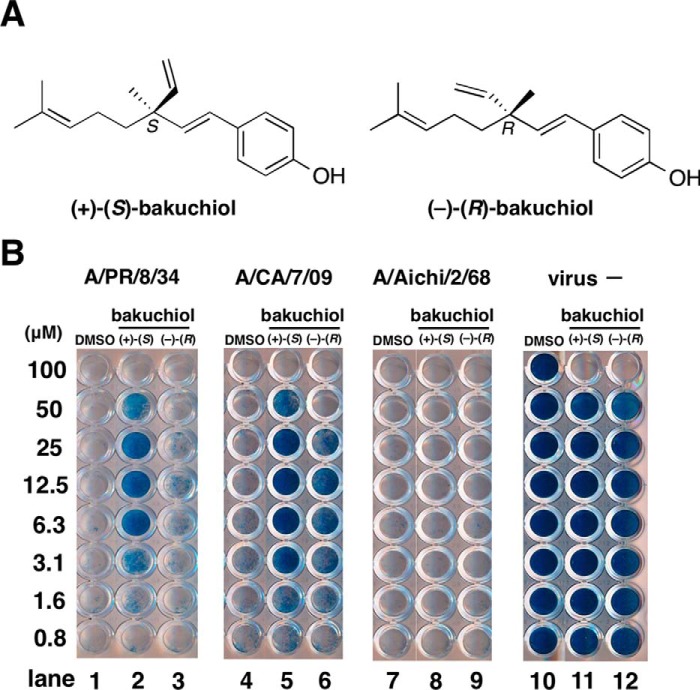
**Effect of bakuchiol on the viability of MDCK cells infected with influenza A virus strains.** *A*, structures of (+)-(*S*)-bakuchiol (naturally occurring) and (−)-(*R*)-bakuchiol (not naturally occurring). *B*, the indicated concentrations of (+)-(*S*)-bakuchiol or (−)-(*R*)-bakuchiol in DMSO were mixed with the indicated influenza A virus strains (MOI = 10) or without influenza A virus (*virus* −) and added to MDCK cells. After incubation for 4 days, the cell viability was determined by naphthol blue black staining. Data are representative of two independent experiments, and the results were reproducible.

An influenza A pandemic caused 50 million deaths worldwide in 1918 (1, 2), the influenza A virus that originated in swine (H1N1) caused a pandemic in 2009, and avian H5N1 and H7N9 influenza A viruses are highly pathogenic to humans (1–3). Although neuraminidase (NA)^2^ inhibitors of the influenza virus have been widely used as antiviral drugs (4, 5), adverse effects (6–9) and the emergence of resistant viral strains (10, 11) have been reported. Thus, to prevent and to control future influenza epidemics and pandemics, it is critically important that novel anti-influenza drugs be developed.

(+)-(*S*)-Bakuchiol is a naturally occurring phenolic isoprenoid (Fig. 1*A*) with a chiral tetra-alkylated (all-carbon) quaternary center that has been isolated from the seeds of *Psoralea corylifolia* L.; this plant (known as Babchi) is used in Chinese and Indian traditional medicine systems to treat a range of diseases (12), such as inflammation due to *Acne vulgaris* (13), breast and lung cancers (14, 15), bone loss (16), neurological disorders (17), and oxidative stress (18). (+)-(*S*)-Bakuchiol can be chemically synthesized from (*E*)-geranic acid in four steps (19). Bakuchiol has been reported to possess a range of biological and pharmacological activities, including anti-microbial (20), antioxidant (21, 22), anti-inflammatory (23, 24), and anti-tumor (15, 25) effects. Bakuchiol was shown to inhibit mitochondrial lipid peroxidation (21) and to induce apoptosis in human lung adenocarcinoma A549 cells via reactive oxygen species (ROS)-dependent disruption of mitochondrial membrane potential (15). Based on these results, we aimed to determine whether bakuchiol also possessed anti-influenza virus activity.

In the present study, we found that (+)-(*S*)-bakuchiol and (−)-(*R*)-bakuchiol (a synthetic enantiomer that does not occur naturally; Fig. 1*A*) inhibited influenza A H1N1 viral infection and growth in Madin-Darby canine kidney (MDCK) cells and also reduced the expression of viral mRNAs and proteins. They reduced the induction of interferon-β (*IFN-*β) and myxovirus-resistant protein 1 (*Mx1*) mRNAs by the influenza A virus. (+)-(*S*)-Bakuchiol showed stronger antiviral activities than (−)-(*R*)-bakuchiol, indicating that the steric structure was important for these activities. We used an influenza A virus minigenome assay employing a Dual-Luciferase system to analyze mRNA and protein levels, and this unexpectedly revealed that bakuchiol induced host factors that inhibited firefly and *Renilla* luciferases. Next generation sequencing (NGS) and KeyMolnet analysis revealed an up-regulation of transcriptional regulation by the nuclear factor erythroid 2-related factor (Nrf) pathway, and a Nrf2 reporter assay showed that (+)-(*S*)-bakuchiol activated Nrf2. Reverse transcription quantitative polymerase chain reaction (RT-qPCR) analyses showed that bakuchiol up-regulated mRNA expression of NAD(P)H quinone oxidoreductase 1 (*NQO1*) and glutathione *S*-transferase A3 (*GSTA3*); these are Nrf2-induced oxidative stress-responsive genes. Taken together, these results indicated that bakuchiol produced novel anti-influenza effects by targeting processes involved in the host oxidative stress response.

## Experimental Procedures

### 

#### 

##### Preparation of (+)-(S)-Bakuchiol and (−)-(R)-Bakuchiol

(+)-(*S*)-Bakuchiol and (−)-(*R*)-bakuchiol were chemically synthesized from geranic acid and purified using methods reported previously (19). Their chemical structures are shown in Fig. 1*A*. Stock solutions (10 mm) were formed by dissolving these compounds in dimethyl sulfoxide (DMSO).

##### Cells

MDCK cells were cultured in growth medium: high-glucose Dulbecco's modified Eagle's medium (DMEM; Wako, Osaka, Japan) supplemented with 10% fetal bovine serum (FBS; Life Technologies, Inc.); 50 units/ml penicillin and 50 μg/ml streptomycin (Life Technologies); and 4 mm l-glutamine, at 37 °C in the presence of 5% CO_2_.

##### Viral Strains

This study used three strains of influenza A virus: Puerto Rico 8/34 (A/PR/8/34; H1N1), California 7/09 (A/CA/7/09; H1N1), and Aichi 2/68 (A/Aichi/2/68; H3N2). The viral titers were determined by immunostaining the influenza A viral nucleoprotein (NP), as described previously (26, 27).

##### Analysis of the Effects of Influenza A Virus on MDCK Viability Using Naphthol Blue Black

MDCK cells were seeded in a 96-well plate (1 × 10^4^ cells/well). (+)-(*S*)-Bakuchiol or (−)-(*R*)-bakuchiol (0.8–100 μm in DMSO) was mixed with an influenza A virus strain (A/PR/8/34, A/CA/7/09, or A/Aichi/2/68) in the growth medium at a multiplicity of infection (MOI) of 10 and then incubated for 30 min at 37 °C under 5% CO_2_. The mixture was added to the cells, and the treated cells were incubated for 4 days at 37 °C under 5% CO_2_. After incubation, the cells were fixed using 10% formaldehyde in phosphate-buffered saline (PBS). The viable cells were then stained with a naphthol blue black solution (0.1% naphthol blue black, 0.1% sodium acetate, and 9% acetic acid) as described previously (28).

##### Thiazolyl Blue Tetrazolium Bromide (MTT) Assay

The toxicities of (+)-(*S*)-bakuchiol and (−)-(*R*)-bakuchiol toward MDCK cells were determined using an MTT cell proliferation assay kit, according to the manufacturer's instructions (Cayman). Briefly, MDCK cells were seeded in each well of a 96-well plate (1 × 10^4^ cells/well). Marchantin E (ME) was used as a positive control for anti-influenza viral activity (27, 28). (+)-(*S*)-Bakuchiol, (−)-(*R*)-bakuchiol, or ME (12.5–100 μm) was prepared in DMSO (100 μm, 1%; 50 μm, 0.5%; 25 μm, 0.25%; 12.5 μm, 0.125%) and mixed with infection medium (DMEM supplemented with 1% bovine serum albumin (BSA; Wako, Osaka, Japan), 50 units/ml penicillin, 50 μg/ml streptomycin, and 4 mm l-glutamine). The mixture was added to the cells, and the treated cells were incubated for 24 or 96 h at 37 °C under 5% CO_2_. After incubation, the cells were treated with the MTT reagent and incubated for 4 h at 37 °C under 5% CO_2_. The wells were then treated with crystal-dissolving solution to dissolve the formazan produced by the cells, and the absorbance of each well was measured at 570 nm using a microplate reader. Cell viability was calculated and expressed relative to that of DMSO-treated cells, which was set as 100%.

##### Immunostaining of Influenza A Virus-infected Cells

MDCK cells were seeded in a 96-well plate (1 × 10^4^ cells/well). (+)-(*S*)-Bakuchiol, (−)-(*R*)-bakuchiol, or ME was mixed at a concentration of 12.5–50 μm with influenza A virus (A/PR/8/34, A/CA/7/09, or A/Aichi/2/68) at an MOI of 0.1 in the infection medium and incubated for 30 min at 37 °C under 5% CO_2_. DMSO (0.125–0.5%) was used as the negative control. Each mixture was added to the cells and incubated for 24 h at 37 °C under 5% CO_2_. The cells were then fixed with 4% paraformaldehyde in PBS for 30 min at 4 °C before permeabilization with 0.3% Triton X-100 for 20 min at room temperature. Mouse antibodies detecting the NP of A/PR/8/34 and A/Aichi/2/68 (FluA-NP 4F1, SouthernBiotech) or the NP of A/CA/7/09 (AA5H, AbD Serotec) were used as primary antibodies, as appropriate (26). Horseradish peroxidase-conjugated goat anti-mouse IgG (SouthernBiotech) was used as the secondary antibody. To visualize the infected cells, TrueBlue peroxidase substrate (KPL) was added and incubated for 15 min; color development was terminated by washing with H_2_O. The wells were photographed under a microscope, and the stained cells were counted. Each half-maximal (50%) inhibitory concentration (IC_50_) value was then calculated based on the cell numbers.

##### Influenza A Viral Infection and Growth Assays

To explore whether bakuchiol preincubation affected viral infection, 1 × 10^5^ MDCK cells were seeded in each well of a 24-well plate. (+)-(*S*)-Bakuchiol or (−)-(*R*)-bakuchiol (25 μm each) were mixed with A/PR/8/34 (MOI = 0.001) in the infection medium supplemented with 3 μg/ml l-tosylamido-2-phenyl ethyl chloromethyl ketone (TPCK)-treated trypsin (Sigma-Aldrich) and incubated for 30 min at 37 °C under 5% CO_2_. DMSO (0.5%) was the negative control, and ME (50 μm) was the positive control. Each mixture was added to the cells and incubated for 24, 48, or 72 h at 37 °C under 5% CO_2_.

To explore whether bakuchiol affected viral growth in preinfected cells, 1 × 10^5^ MDCK cells were seeded in each well of a 24-well plate. The cells were infected with A/PR/8/34 (MOI = 0.001) in the infection medium for 1 h at 37 °C under 5% CO_2_. The infected cells were washed, and then (+)-(*S*)-bakuchiol or (−)-(*R*)-bakuchiol (25 μm) was added to the cells in the infection medium supplemented with 3 μg/ml TPCK-treated trypsin. DMSO (0.5%) was the negative control, and ME (50 μm) was the positive control. The cells were then incubated for 24, 48, or 72 h at 37 °C under 5% CO_2_.

Cell culture media were collected from each well at the indicated time points. Serial dilutions of the conditioned media were added to naive monolayers of MDCK cells in a 96-well plate and incubated for 16 h at 37 °C under 5% CO_2_. The cells were immunostained using FluA-NP 4F1 (SouthernBiotech), as described above (26), and the stained cells were counted. The viral titers in the conditioned media were calculated using these cell numbers (26).

##### RT-qPCR

Total RNA was extracted from MDCK cell lysates using an RNeasy minikit (Qiagen, GmbH, Hilden, Germany). Total RNA (500 ng) was used to synthesize cDNA using SuperScript VILO (Invitrogen), according to the manufacturer's instructions. The synthesized cDNA was used as a template for RT-qPCR, which was performed using SYBR Green real-time PCR Master Mix (TOYOBO, Osaka, Japan); each gene-specific primer employed is shown in supplemental Table 1. PCR and data analyses were performed on an Applied Biosystems StepOne Plus Real-time PCR system (Life Technologies). Relative expression was calculated by the ΔΔ*Ct* method. The levels of viral mRNAs encoding nonstructural protein 1 (*NS1*), *NP*, RNA polymerase subunits (*PA*, *PB1*, and *PB2*), and matrix genes (*M1* and *M2*) were normalized to that of 18S ribosomal RNA (rRNA) (29), and the levels of *IFN*-β, *Mx1*, *NQO1*, *GSTA3*, firefly luciferase, and *Renilla* luciferase mRNAs were normalized to that of β-actin.

##### Western Blotting

The cells were lysed in a buffer containing 125 mm Tris-HCl, pH 6.8, 5% SDS, 25% glycerol, 0.1% bromphenol blue, and 10% β-mercaptoethanol and boiled for 5 min. The cell lysates were then separated on a 10% polyacrylamide gel. The proteins were transferred to a polyvinylidene fluoride microporous membrane (Millipore). FluA-NP 4F1 (SouthernBiotech), a goat anti-influenza A viral NS1 antibody (vC-20, Santa Cruz Biotechnology, Inc.), a rabbit anti-firefly luciferase polyclonal antibody (MBL, Nagoya, Japan), and a rabbit anti-*Renilla* luciferase polyclonal antibody (MBL) were used as primary antibodies to detect their respective proteins. A rabbit anti-β-actin antibody (13E5, Cell Signaling) was used as an internal control. The secondary antibodies, horseradish peroxidase-conjugated goat anti-mouse IgG (SouthernBiotech), donkey anti-goat IgG (sc-2020, Santa Cruz Biotechnology), or goat anti-rabbit IgG (KPL), were used as appropriate. The signals were detected using Western Lightning ECL Pro (PerkinElmer Life Sciences). Signal intensities were measured using ImageJ software, and the protein levels of firefly and *Renilla* luciferase were normalized to that of β-actin.

##### NA Assay with Influenza A Viral NA Protein or Viral Particles

NA assays were performed as described previously (30). Briefly, (+)-(*S*)-Bakuchiol or (−)-(*R*)-bakuchiol (12.5–50 μm) were diluted with assay buffer (50 mm Tris, 5 mm CaCl_2_, and 200 mm NaCl, pH 7.5) in a 96-well black plate (Nunc, Thermo Scientific). DMSO (0.125–0.5%) was used as the negative control and oseltamivir carboxylate (9) (12.5–50 μm) was the positive control. Two nanograms of recombinant influenza A virus H1N1 NA protein (R&D Systems) or influenza A virus (A/PR/8/34 or A/CA/7/09 at 1 × 10^4^ pfu) in assay buffer were added to each well and incubated for 30 min at 37 °C under 5% CO_2_. Each sample was mixed with 12.5 μm 2′-(4-methylumbelliferyl)-α-d-*N*-acetylneuraminic acid (Sigma-Aldrich) in a 96-well plate. After 0, 3, or 24 h at 37 °C under 5% CO_2_, the reaction was monitored in a fluorescence spectrometer in a kinetic mode using an excitation wavelength of 365 nm and an emission wavelength of 445 nm.

##### Hemagglutination Assay

A/PR/8/34 and A/CA/7/09 were serially diluted with PBS to achieve 0.08–10 × 10^4^ pfu in a round-bottomed 96-well plate and incubated for 30 min at 37 °C under 5% CO_2_. Chicken red blood cells (KOHJIN BIO, Saitama, Japan) were added to each well at a concentration of 0.5% in PBS. After incubation for 1 h at room temperature, the plate was photographed. Next, (+)-(*S*)-bakuchiol or (−)-(*R*)-bakuchiol was diluted (0.8–100 μm) with PBS in a round-bottomed 96-well plate. DMSO (0.008–1%) was used as a negative control. A/PR/8/34 (0.63 or 0.31 × 10^4^ pfu) or A/CA/7/09 (1.25 or 0.63 × 10^4^ pfu) in PBS was added to each well and incubated for 30 min at 37 °C under 5% CO_2_. Each sample was mixed with 0.5% chicken red blood cells in PBS. After incubation for 1 h at room temperature, the plates were photographed.

##### Trypsin Protection Assay with Influenza A Viral Hemagglutinin (HA)

The HA protein trypsin protection assay was performed as described previously (31). Recombinant influenza A virus (A/PR/8/34) HA protein (0.5 μg) (Sino Biological Inc., Beijing, China) was incubated with DMSO (0.25% in PBS, adjusted to pH 5.0 with 0.25 m HCl) for 30 min at 31 °C. The pH of the reaction was then neutralized to a final pH of 7.5 using 0.25 m NaOH. TPCK-treated trypsin (0.0001–1 μg) (Sigma-Aldrich) was added to each mixture and digested for 30 min at 37 °C. Trypsin-mediated HA cleavage was determined by SDS-PAGE followed by staining with Coomassie Blue G-250. Next, 0.5 μg of recombinant influenza A virus (A/PR/8/34) HA protein (Sino Biological Inc.) was incubated with (+)-(*S*)-bakuchiol or (−)-(*R*)-bakuchiol (25–100 μm) in PBS for 15 min at 37 °C and then adjusted to a final pH of 5.0 with 0.25 m HCl and incubated for a further 15 min at 31 °C. The pH was then neutralized to 7.5 using 0.25 m NaOH. TPCK-trypsin (Sigma-Aldrich) (1 μg in PBS) was added to each mixture and digested for 30 min at 37 °C. Trypsin-mediated HA cleavage was determined by SDS-PAGE followed by staining with Coomassie Blue G-250.

##### Influenza A Viral M2 Channel Activity

Using the whole-cell patch clamp technique, M2 channel currents were recorded from Chinese hamster ovary (CHO-K1) cells that had been transfected for 24–48 h with pCA-M2 encoding a M2 cDNA cloned from influenza virus A/PR/8/34 together with pCA-GFP, encoding the green fluorescent protein (GFP) gene. Gene expression was driven by the chicken β-actin promoter with the cytomegalovirus enhancer (CA). Transfected cells were plated on a coverslip, placed in a recording chamber fixed to the microscope stage, and perfused with a solution containing 135 mm *N*-methyl-d-glucamine, 25 mm HEPES, 5 mm CaCl_2_, and 10 mm glucose (pH adjusted to 7.4 with 1 n HCl). Recordings were made at a flow rate of 1.0–1.5 ml/min at room temperature (27–28 °C). Cells expressing M2 channels were identified using confocal laser-scanning microscopy (LSM510, Carl Zeiss, Jena, Germany) by detecting the co-expressed GFP fluorescence at an excitation wavelength of 488 nm. Borosilicate glass capillaries (1B150F-4, World Precision Instruments, Inc.) were used to produce patch electrodes using a Flaming/Brown micropipette puller (P-97, Sutter Instruments). The electrode had a resistance of 2–5 megaohms when filled with a pipette solution of 90 mm *N*-methyl-d-glucamine, 10 mm EGTA, and 180 mm MES, with the pH adjusted to 6.0 using 1 n HCl. Membrane currents were recorded from cells held at −40 mV using Multiclamp 700B (Axon Instruments) via a Digidata 1322A interface (Axon Instruments) and stored on a computer hard disk with Clampex version 9.2 software (Axon Instruments). M2 current data were analyzed by Clampfit version 9.2 (Axon Instruments). M2 currents were induced by exposing CHO-K1 cells to a brief puff (duration, 0.1–1 s) of a low-pH solution (135 mm *N*-methyl-d-glucamine, 25 mm MES, 5 mm CaCl_2_, and 10 mm glucose, pH adjusted to 6.0 with HCl) every 20 s via a glass micropipette using a Pneumatic PicoPump (PV830, World Precision Instruments). Drugs were dissolved in the external control solution (pH 7.4) and applied by perfusion after a control period (3 min), during which M2 currents with stable amplitudes were obtained. (+)-(*S*)-Bakuchiol (20 or 50 μm) was dissolved in control solution supplemented with 0.1% DMSO to aid dissolution and 0.5% BSA to prevent adsorption to the perfusion lines. Amantadine (100 μm) was used as the positive control.

##### Minigenome Assay

A minigenome assay based on the Dual-Luciferase system was performed as described previously (32, 33). The plasmids pCA-PA, pCA-PB1, pCA-PB2, and pCA-NP included the influenza PA, PB1, PB2, and NP genes, respectively, driven by CA. pPolI/NP(0)Fluc(0) expresses the minus RNA strand of firefly luciferase driven by the vRNA promoter; this can be converted to the plus strand (mRNA) by viral RNA-dependent RNA polymerase (RdRp). The pRL-TK-Rluc vector (Promega) expresses *Renilla* luciferase driven by the herpes simplex viral thymidine kinase promoter and was used as an internal control. MDCK cells (5 × 10^4^) were transfected with 0.2 μg of pCA-PA, -PB1, -PB2, or -NP, with pPolI/NP(0)Fluc(0) (0.2 μg) and pRL-TK-Rluc (0.2 μg). At 24 h post-transfection, the cells were treated with 50 μm (+)-(*S*)-bakuchiol or (−)-(*R*)-bakuchiol at 37 °C under 5% CO_2_. DMSO (0.5%) or 50 μm ribavirin (MP Biomedicals, Illkirch, France) was used as the negative or positive control, respectively. After a 24-h incubation, the luciferase activity in the transfected MDCK cells was measured using the Dual-Glo luciferase assay system (Promega), according to the manufacturer's instructions. Each luciferase activity was expressed as relative light units (RLU), where the activity in the DMSO-treated (negative control) cells represented 100%.

##### Analyses of Reporter Assays and Protein Levels of Firefly and Renilla Luciferases

MDCK cells were seeded in a 96-well plate (1 × 10^4^ cells/well). The cells were transfected with pGL3-control (Promega, 0.1 μg), expressing firefly luciferase driven by the SV40 promoter, and pRL-TK-Rluc (0.1 μg). At 24 h post-transfection, the cells were treated with 1, 5, 25, or 50 μm (+)-(*S*)-bakuchiol or (−)-(*R*)-bakuchiol or with 50 μm ribavirin at 37 °C under 5% CO_2_. DMSO (0.5%) was used as a negative control. After a 24-h incubation, luciferase activity in the transfected MDCK cells was measured using the Dual-Glo luciferase assay system, and the levels of firefly and *Renilla* luciferase protein were also measured by Western blotting.

##### Transcriptome Analysis by NGS

We used NGS to conduct a comprehensive transcriptome analysis in MDCK cells treated with bakuchiol and influenza virus (A/PR/8/34), using the method previously reported by Kanematsu *et al.* (34). Briefly, 1 × 10^5^ MDCK cells were seeded in each well of a 24-well plate. (+)-(*S*)-Bakuchiol or (−)-(*R*)-bakuchiol (25 μm) were mixed with A/PR/8/34 at an MOI of 0.1. DMSO (0.25%) was used as a negative control. Each mixture was added to the MDCK cells and incubated for 24 h before extracting total RNA from the cell lysates. mRNA-sequencing libraries were constructed from each total RNA extract using the SureSelect strand-specific RNA library preparation kit (Agilent Technologies), according to the manufacturer's instructions. Thirty-six-base pair, single-end-read RNA sequencing tags were generated using an Illumina Hiseq2500 sequencer (Illumina). RNA sequencing tags that mapped to the dog reference genome sequences (CanFam3 genome) were analyzed. The reads per kilobase per million mapped reads (RPKM) were calculated for the mRNA transcripts in Ensemble. Genes that showed a >1.5-fold change in RPKM value in (+)-(*S*)-bakuchiol-treated MDCK cells are indicated in supplemental Table 2. The complete NGS transcriptome analysis has been deposited in the DNA Data Bank of Japan database (accession number DRA003499) and in the Gene Expression Omnibus (accession number GSE73750).

##### Molecular Network and Pathway Analysis

The molecular networks and pathways in the NGS analysis were analyzed by the KeyMolnet Viewer program version 5.9, *in silico* (35).

##### Nrf2 Reporter Assay

An Nrf2 reporter assay based on the Dual-Luciferase system was performed as described previously (36). The plasmid, pNQO1-ARE (antioxidant response element)-luc expresses a firefly luciferase gene driven by Nrf2 activation (36), and pRL-TK-Rluc was used as an internal control. MDCK cells (1 × 10^5^) were seeded in each well of a 24-well plate and transfected with pNQO1-ARE-luc (0.25 μg) and pRL-TK-Rluc (0.25 μg). At 24 h post-transfection, the cells were treated with 25 μm (+)-(*S*)-bakuchiol or (−)-(*R*)-bakuchiol in the infection medium at 37 °C under 5% CO_2_. DMSO (0.25%) or 25 μm dl-sulforaphane (Sigma-Aldrich), which enhances Nrf2-driven gene expression (37), were used as the negative and positive control, respectively. Total RNA was extracted from the MDCK cell lysates after a 24-h incubation. The levels of firefly and *Renilla* luciferase mRNA were analyzed by RT-qPCR, normalized to β-actin mRNA.

##### Statistical Analysis

All results were expressed as the mean ± S.E. Differences between two groups were analyzed for statistical significance by Student's *t* test, whereas those between more than two groups were analyzed by one-way analysis of variance. The results were considered significantly different when *p* was <0.05.

## Results

### 

#### 

##### Bakuchiol Increased the Survival of Infected MDCK Cells

**FIGURE 2.**
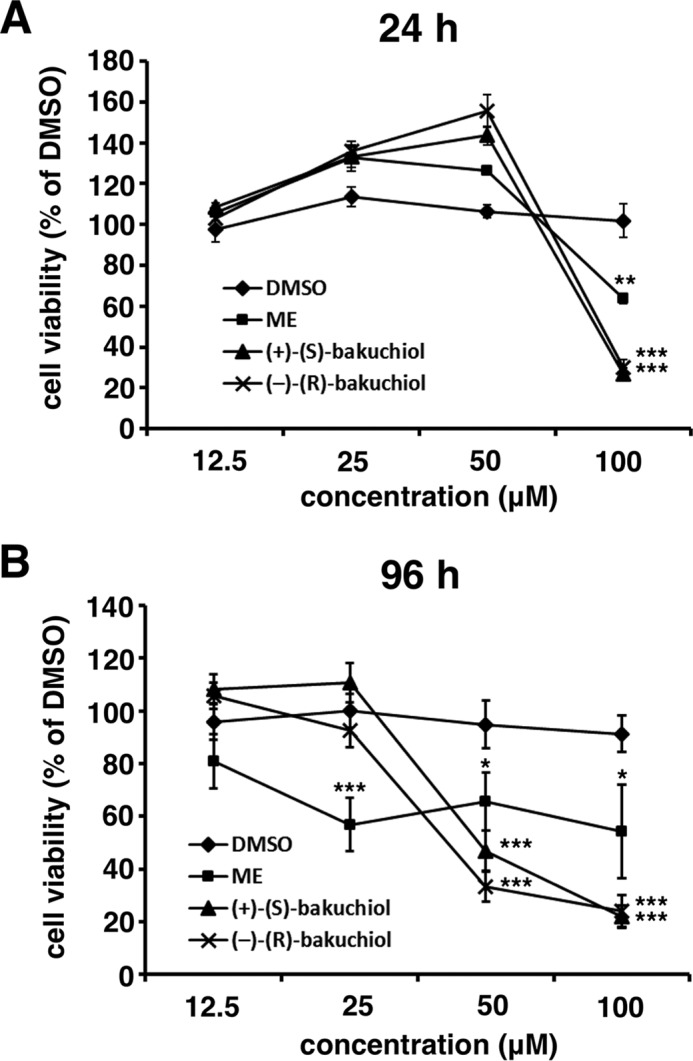
**Toxicity of bakuchiol against MDCK cells.** The indicated concentrations of (+)-(*S*)-bakuchiol, (−)-(*R*)-bakuchiol, or ME in DMSO (concentrations of DMSO: 100 μm, 1%; 50 μm, 0.5%; 25 μm, 0.25%; and 12.5 μm, 0.125%) were added to the MDCK cells. Cell viabilities were determined by MTT assay after 24 h (*n* = 4 each) (*A*) or 96 h (*n* = 6 each) (*B*). Data represent the mean ± S.E. (*error bars*) and are representative of three independent experiments. **, *p* < 0.01; ***, *p* < 0.001 for the comparison with DMSO.

To evaluate the anti-influenza virus activity of bakuchiol, we first examined its effect on the survival of influenza A virus-infected MDCK cells (15). Various concentrations of (+)-(*S*)-bakuchiol or (−)-(*R*)-bakuchiol were mixed with A/PR/8/34, A/CA/7/09, or A/Aichi/2/68 (MOI = 10) and added to MDCK cells. After incubation for 4 days, the cells were stained by naphthol blue black. Viable cells in each well were stained blue, whereas dead cells remained clear. As shown in Fig. 1*B*, cells exposed to DMSO and infected with A/PR/8/34, A/CA/7/09, or A/Aichi2/68 were not stained. However, cells treated with 3.1–50 μm (+)-(*S*)-bakuchiol and infected with A/PR/8/34 or with 1.6–50 μm (+)-(*S*)-bakuchiol and infected with A/CA/7/09 were stained blue. Cells exposed to (−)-(*R*)-bakuchiol and A/PR/8/34 were not stained (Fig. 1*B*), and those infected with A/CA/7/09 in the presence of 3.1–25 μm (−)-(*R*)-bakuchiol showed weak staining (Fig. 1*B*). A/Aichi2/68-infected cells treated with DMSO, (+)-(*S*)-bakuchiol, and (−)-(*R*)-bakuchiol were not stained (Fig. 1*B*, *lanes 7–9*). Cells that were not infected and exposed to 100 μm (+)-(*S*)-bakuchiol and (−)-(*R*)-bakuchiol were not stained (Fig. 1*B*); these findings indicated that 100 μm, but not ≤50 μm, bakuchiol was toxic to MDCK cells in the growth medium. To evaluate the exact cytotoxicity, we analyzed MDCK cells following 24- or 96-h incubations in infection medium containing BSA (instead of FBS) using the MTT assay (Fig. 2). The viability of MDCK cells treated with 100 μm (+)-(*S*)-bakuchiol or (−)-(*R*)-bakuchiol was reduced after 24 or 96 h (Fig. 2, *A* and *B*), as compared with those exposed to DMSO only, whereas the viability of cells exposed to ≤50 or 25 μm bakuchiol was not affected at 24 h or 96 h, respectively (Fig. 2, *A* and *B*). This suggested that exposure to ≤50 or 25 μm bakuchiol for 24 or 96 h did not induce apoptosis in MDCK cells. Therefore, these data showed that (+)-(*S*)-bakuchiol promoted the survival of MDCK cells infected with the influenza A virus H1N1 strain but not of those infected with the H3N2 strain.

##### Bakuchiol Inhibited Influenza A Virus H1N1 Infection and Growth

**FIGURE 3.**
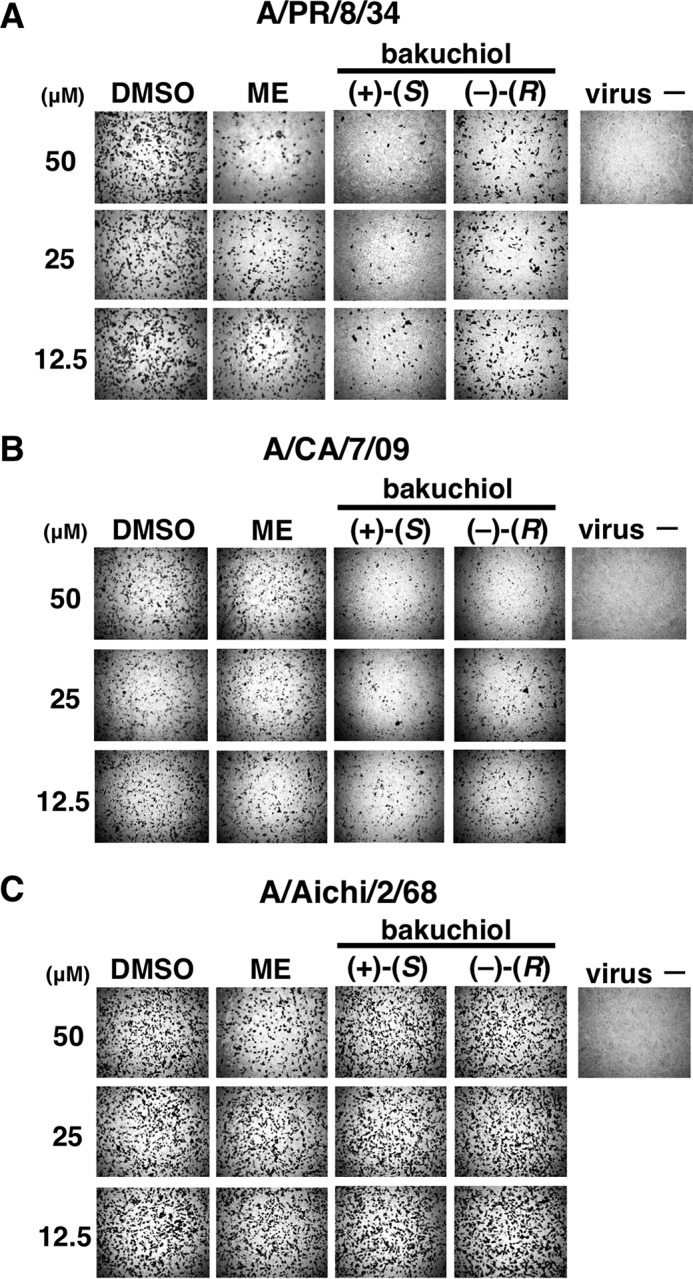
**Bakuchiol inhibited influenza A virus H1N1 infection.** The indicated concentrations of (+)-(*S*)-bakuchiol, (−)-(*R*)-bakuchiol, ME (positive control), or DMSO (0.125–0.5% as the negative control) were mixed with the indicated influenza A virus strains (MOI = 0.1) and added to MDCK cells for 24 h. To visualize infected cells, the MDCK cells were then immunostained with an antibody to influenza A viral NP and photographed under a microscope (*A–C*).

**FIGURE 4.**
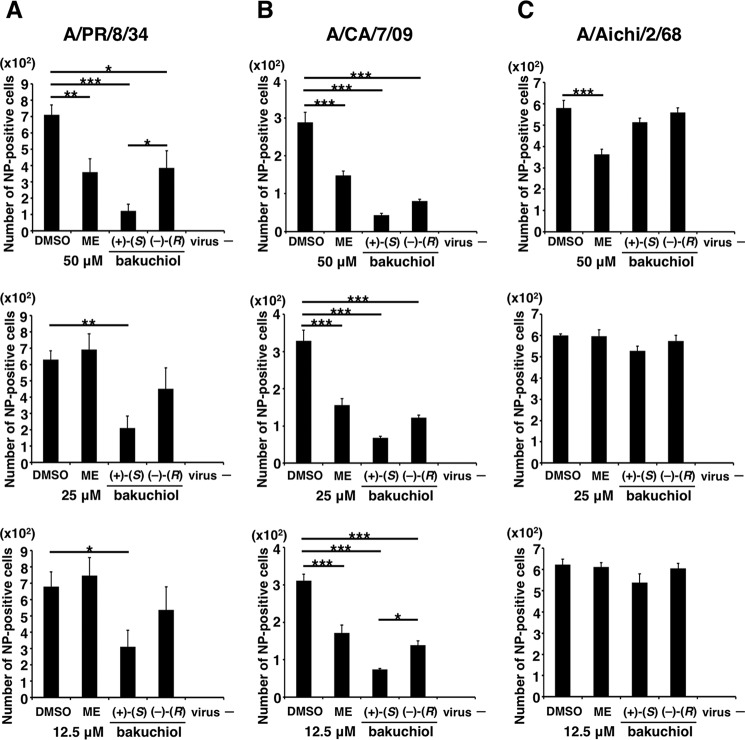
**Bakuchiol inhibited influenza A virus H1N1 infection (number of infected cells).** The indicated concentrations of (+)-(*S*)-bakuchiol (*n* = 5), (−)-(*R*)-bakuchiol (*n* = 5), ME (positive control; *n* = 4), or DMSO as the negative control (0.125–0.5%; *n* = 4) were mixed with the indicated influenza A virus strains (MOI = 0.1) and added to MDCK cells for 24 h. The stained cells indicated in Fig. 3 were counted (*A–C*). Data are presented as the mean ± S.E. (*error bars*) of two independent experiments. *, *p* < 0.05; **, *p* < 0.01; ***, *p* < 0.001 for the indicated comparisons.

To investigate whether bakuchiol inhibited influenza A viral infection, we examined influenza A viral NP-immunostaining in MDCK cells treated with a mixture of influenza A virus (A/PR/8/34, A/CA/7/09, or A/Aichi/2/68; MOI = 0.1) and bakuchiol (12.5–50 μm) or ME for 24 h. The wells were observed under the microscope and photographed (Fig. 3, *A–C*), and the immunostained cells were counted (Fig. 4, *A–C*). The numbers of NP-stained cells were significantly decreased in cells treated with (+)-(*S*)-bakuchiol, (−)-(*R*)-bakuchiol, or ME (positive control) and infected with A/PR/8/34 or A/CA/7/09, as compared with DMSO-treated cells (Fig. 4, *A* and *B*). The number of stained cells in (+)-(*S*)-bakuchiol-treated wells was lower than that observed in wells containing (−)-(*R*)-bakuchiol (Fig. 4, *A* and *B*). In cells infected with A/Aichi/2/68, the numbers of stained cells in (+)-(*S*)-bakuchiol- or (–)-(*R*)-bakuchiol-treated wells were equal to those observed in DMSO-treated wells (Fig. 4*C*). The number of NP-stained cells in wells exposed to 50 μm ME and infected with A/Aichi/2/68 was significantly lower than that observed in DMSO-treated wells (Fig. 4*C*). The IC_50_ values for (+)-(*S*)-bakuchiol against A/PR/8/34 and A/CA/7/09 were 13.9 and 0.2 μm, respectively, whereas the corresponding values for (−)-(*R*)-bakuchiol were 137.7 and 7.5 μm, respectively (Table 1). The IC_50_ values for the positive control (ME) against A/PR/8/34, A/CA/7/09, and A/Aichi/2/68 were 27.9, 13, and 24.8 μm, respectively (Table 1). Therefore, these data showed that bakuchiol had an enantiomer-specific inhibitory effect on influenza A virus H1N1 infection.

**TABLE 1:** **Antiviral effects of bakuchiol against influenza A virus H1N1 and H3N2 strains** Data represent the mean ± S.E. half maximal (50%) inhibitory concentration (IC_50_) values and are representative of two independent experiments. ND, not detected.

	Influenza A virus strains		
	A/PR/8/34; H1N1	A/CA/7/09; H1N1	A/Aichi/2/68; H3N2
Marchantin E (μm)	27.9 ± 1.8	13.0 ± 2.9	24.8 ± 0.5
(+)-(*S*)-bakuchiol (μm)	13.9 ± 6.1	0.2 ± 0.1	ND
(−)-(*R*)-bakuchiol (μm)	137.7 ± 91.7	7.5 ± 4.2	ND

**FIGURE 5.**
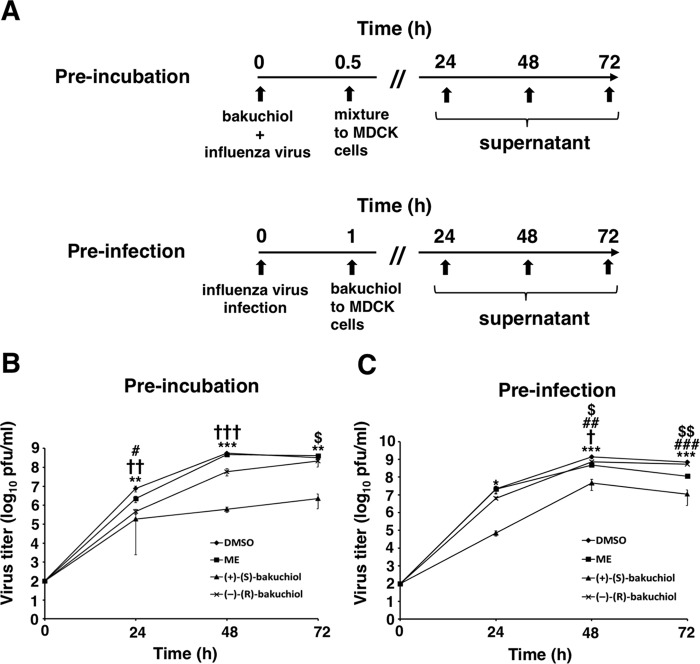
**Bakuchiol inhibited influenza A virus H1N1 infection and growth.** *A*, schematic showing the preincubation and preinfection protocols using the A/PR/8/34 (H1N1) strain (MOI = 0.001). In both approaches, 25 μm (+)-(*S*)-bakuchiol (*n* = 4) or (−)-(*R*)-bakuchiol (*n* = 4) was used as indicated. For preincubation, bakuchiol was mixed with A/PR/8/34 supplemented with 3 μg/ml TPCK-treated trypsin before the addition to MDCK cells. DMSO (0.5%; *n* = 4) was the negative control, and 50 μm ME (*n* = 4) was the positive control. For preinfection, MDCK cells were infected with A/PR/8/34 before the addition of bakuchiol supplemented with 3 μg/ml TPCK-treated trypsin. DMSO (0.25%; *n* = 4) and 50 μm ME (*n* = 4) were the negative and positive controls, respectively. *B* and *C*, the conditioned culture media were collected at the indicated time points and added to MDCK cells, and the treated cells were immunostained with an antibody to influenza A viral NP. The viral titers were calculated from the number of stained cells. Data represent the mean ± S.E. (*error bars*) and are representative of two independent experiments. *, *p* < 0.05; **, *p* < 0.01; ***, *p* < 0.001 for the comparison of DMSO and (+)-(*S*)-bakuchiol. †, *p* < 0.05; ††, *p* < 0.01; †††, *p* < 0.001 for the comparison of DMSO and (−)-(*R*)-bakuchiol. #, *p* < 0.05; ##, *p* < 0.01; ###, *p* < 0.001 for the comparison of DMSO and ME. $, *p* < 0.05; $$, *p* < 0.01 for the comparison of (+)-(*S*)-bakuchiol and (−)-(*R*)-bakuchiol.

Next, to investigate whether bakuchiol inhibited influenza A virus H1N1 infection and growth in MDCK cells, we investigated the effects of both enantiomers on viral infection and growth (Fig. 5*A*). The bakuchiol concentration used in these 24–72-h experiments was 25 μm, which did not show cytotoxicity following 96-h exposure to MDCK cell in infection medium (Fig. 2*B*). The preincubation experiment (Fig. 5*A*) involved mixing (+)-(*S*)-bakuchiol, (−)-(*R*)-bakuchiol, or ME with A/PR/8/34 and adding this mixture to MDCK cells (Fig. 5*B*) in order to evaluate whether bakuchiol inhibited viral attachment to these cells. The preinfection experiment (Fig. 5*A*) examined the effects of these treatments in A/PR/8/34-infected MDCK cells (Fig. 5*C*) in order to evaluate whether bakuchiol inhibited viral growth. In both of these approaches, the viral titers in conditioned media from cells treated with (+)-(*S*)-bakuchiol were significantly decreased at 24–72 h, as compared with those in media conditioned by DMSO-treated cells (Fig. 5, *B* and *C*). The viral titers in conditioned media from cells treated with (−)-(*R*)-bakuchiol were significantly decreased at 24 and 48 h, as compared with media conditioned by DMSO-treated cells, whereas these titers showed no significant difference at 72 h (Fig. 5, *B* and *C*). In addition, the viral titers in culture media conditioned by cells treated with (+)-(*S*)-bakuchiol for 48 or 72 h using both the preincubation and preinfection approaches were significantly decreased, as compared with those of media from cells exposed to (−)-(*R*)-bakuchiol (Fig. 5, *B* and *C*). The viral titers in culture media conditioned by cells treated with ME were significantly decreased in both preincubation (24 h) and preinfection (48 and 72 h) experiments, as compared with those in media from DMSO-treated cells (Fig. 5, *B* and *C*). These data showed that bakuchiol inhibited the growth of the influenza A virus H1N1 strain. Taken together, these findings demonstrated that bakuchiol had enantiomer-specific inhibitory effects on influenza A viral infection and growth.

##### Bakuchiol Reduced Expression of Influenza A Virus H1N1 mRNAs and Proteins

**FIGURE 6.**
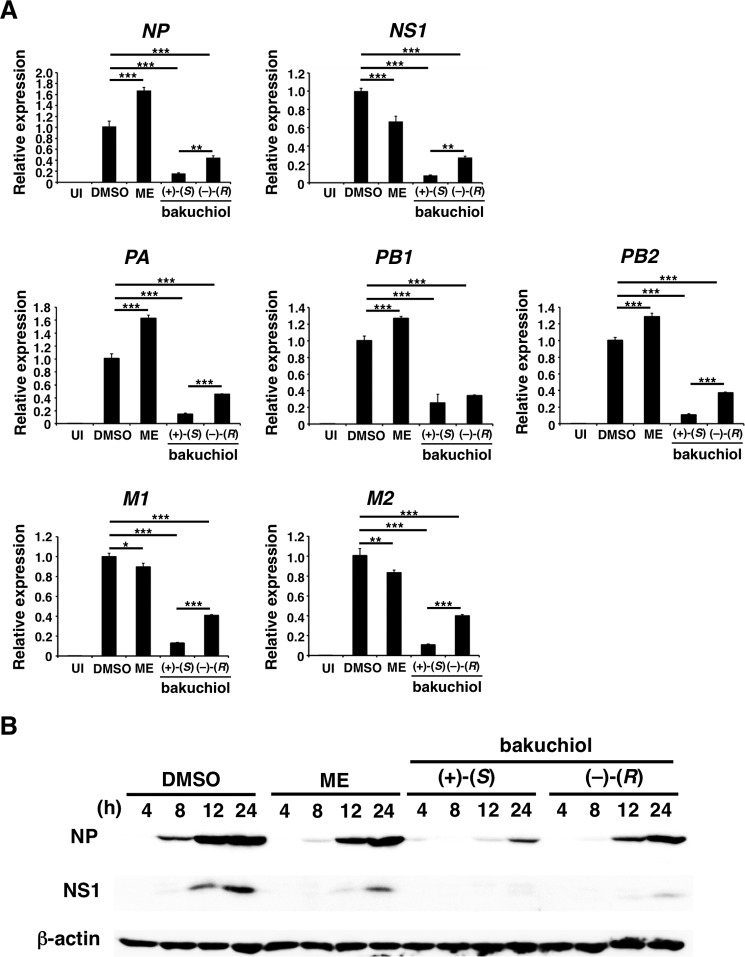
**Bakuchiol inhibited expression of influenza A virus H1N1 mRNAs and proteins.** (+)-(*S*)-Bakuchiol (25 μm), (−)-(*R*)-bakuchiol (25 μm), ME (50 μm; positive control), or DMSO (0.5%; negative control) was mixed with A/PR/8/34 (MOI of 0.1 for mRNA analyses or 1 for protein analyses) and incubated for 30 min before the addition to 1 × 10^5^ MDCK cells. *A*, total RNA was extracted from cell lysates 24 h postinfection, and the relative expression levels of viral mRNAs (*NP*, *NS1*, *PA*, *PB1*, *PB2*, *M1*, and *M2*) (*n* = 4) were determined by RT-qPCR, normalized to 18S ribosomal RNA, and expressed in relation to the levels in the DMSO-treated cells (set as 1). Data represent the mean ± S.E. (*error bars*) and are representative of two independent experiments. *UI*, uninfected cells. *, *p* < 0.05; **, *p* < 0.01; ***, *p* < 0.001. *B*, at 4, 8, 12, or 24 h postinfection, the levels of influenza A viral NP and NS1 proteins in cell lysates were analyzed by Western blotting, and β-actin was analyzed as an internal control. Data are representative of two independent experiments, and the results were reproducible.

To evaluate whether bakuchiol inhibited the expression of influenza A virus H1N1 mRNAs and proteins, we performed RT-qPCR and Western blotting in MDCK cells treated with a mixture of A/PR/8/34 (MOI = 0.1) and bakuchiol or ME for 24 h before the extraction of total RNA and cDNA synthesis. Relative mRNA expression levels of viral genes (*NP*, *NS1*, *PA*, *PB1*, *PB2*, *M1*, and *M2*), analyzed by RT-qPCR, were significantly decreased in MDCK cells treated with (+)-(*S*)-bakuchiol or (−)-(*R*)-bakuchiol, as compared with the levels in DMSO-treated cells (Fig. 6*A*). This reduction was significantly greater in cells treated with (+)-(*S*)-bakuchiol, as compared with those treated with (−)-(*R*)-bakuchiol (Fig. 6*A*). Next, we examined the time course of these effects. (+)-(*S*)-Bakuchiol, (−)-(*R*)-bakuchiol, or ME was mixed with A/PR/8/34 (MOI = 1) and added to MDCK cells. The levels of influenza A viral NP and NS1 proteins were analyzed in lysates of the treated cells by Western blotting after incubation for 4, 8, 12, and 24 h. The levels of NP and NS1 proteins were reduced in MDCK cells treated with (+)-(*S*)-bakuchiol or (−)-(*R*)-bakuchiol for 8–24 h, as compared with the levels in cells treated with DMSO (Fig. 6*B*), and (+)-(*S*)-bakuchiol produced a greater reduction than (−)-(*R*)-bakuchiol. Therefore, these data showed that bakuchiol reduced the expression of influenza A virus H1N1 mRNAs and proteins in a chiral-selective manner.

##### Bakuchiol Reduced the Expression of Host Cell IFN-β and Mx1 mRNA following Viral Infection

**FIGURE 7.**
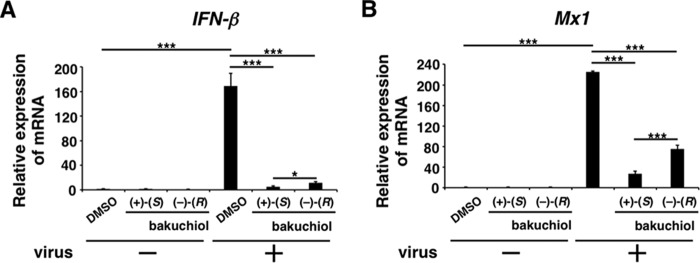
**Bakuchiol inhibited mRNA expression of *IFN*-β and *Mx1* in influenza A virus-infected cells.** (+)-(*S*)-Bakuchiol (25 μm), (−)-(*R*)-bakuchiol (25 μm), or DMSO (0.25%; negative control) was mixed with A/PR/8/34 (MOI = 0.1) and incubated for 30 min before the addition to 1 × 10^5^ MDCK cells. Total RNA was extracted from cell lysates 24 h postinfection. Relative levels of *IFN*-β (*n* = 6) (*A*) and *Mx1* (*n* = 3) (*B*) mRNA were determined by RT-qPCR, normalized to β-actin mRNA, and expressed relative to the levels in DMSO-treated non-infected cells (set as 1). Data are presented as the mean ± S.E. (*error bars*) of three independent experiments. *, *p* < 0.05; **, *p* < 0.01; ***, *p* < 0.001 for the indicated comparisons (*A*; Student's *t* test for the comparison between (+)-(*S*)-bakuchiol and (−)-(*R*)-bakuchiol groups).

Based on our findings indicating that bakuchiol inhibited the infection and growth of the influenza A virus H1N1, we hypothesized that bakuchiol may reduce the host cell immune response induced by this virus. It has previously been reported that influenza A viral infection induced the expression of IFN-β and Mx1 in host cells (38–41). We therefore analyzed the *IFN*-β and *Mx1* mRNA levels in MDCK cells infected with A/PR/8/34 (MOI = 0.1) and treated with bakuchiol. Total RNA was extracted from cell lysates, and the relative expression levels of *IFN*-β and *Mx1* mRNA were analyzed by RT-qPCR (Fig. 7, *A* and *B*). Whereas the *IFN-*β and *Mx1* mRNA levels were up-regulated in MDCK cells treated with A/PR/8/34 and DMSO, this host cell response was significantly reduced in the presence of (+)-(*S*)-bakuchiol or (−)-(*R*)-bakuchiol (Fig. 7, *A* and *B*). The inhibitory effect of (+)-(*S*)-bakuchiol was greater than that of (−)-(*R*)-bakuchiol (Fig. 7, *A* and *B*). These data showed that bakuchiol produced a chiral-selective reduction of the host cell immune response induced by influenza A viral infection.

##### Bakuchiol Had No Marked Inhibitory Effects on Influenza Surface Proteins or Channels

As described above, bakuchiol inhibited infection by influenza A virus H1N1 but not by H3N2 (Fig. 1*B*, *lanes 3* and *4*). We therefore investigated the effects of bakuchiol on the influenza surface proteins (NA, HA, and M2), which possess sialidase, hemagglutination, and H^+^ ion channel activity, respectively (42).

**FIGURE 8.**
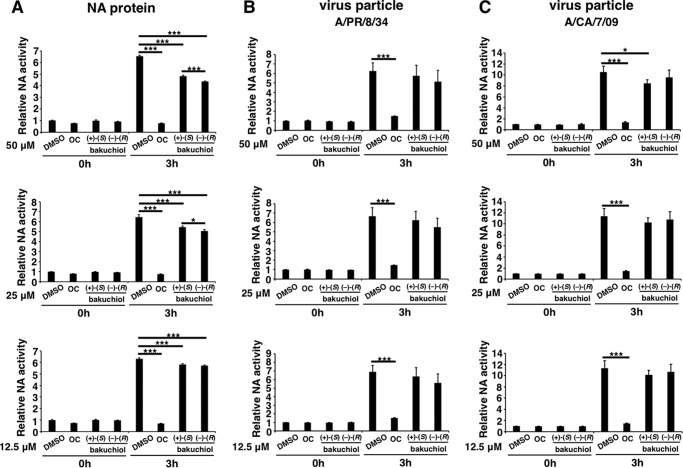
**Influenza A viral NA assay.** Recombinant influenza A virus H1N1 NA protein (*A*) (*n* = 8), influenza virus A/PR/8/34 at an MOI of 0.1 (*B*) (*n* = 3), or influenza virus A/CA/7/09 at an MOI of 0.1 (*C*) (*n* = 3) was added to the indicated concentrations of (+)-(*S*)-bakuchiol, (−)-(*R*)-bakuchiol, oseltamivir carboxylate (*OC*) (positive control), or 0.125–0.5% DMSO (negative control) and mixed with 2′-(4-methylumbelliferyl)-α-d-*N*-acetylneuraminic acid (12.5 μm). At the indicated time points, fluorescence was monitored (excitation, 365 nm; emission, 445 nm). Data represent the mean ± S.E. (*error bars*) and are representative of two or three independent experiments. *, *p* < 0.05; ***, *p* < 0.001 for the indicated comparisons.

To examine the effect of bakuchiol on influenza A H1N1 viral sialidase, we performed the NA assay with H1N1 NA protein or particles (A/PR/8/34 and A/CA/7/09). We showed that oseltamivir carboxylate, an NA inhibitor (9), strongly inhibited the sialidase activity of NA protein (Fig. 8*A*) and viral particles (Fig. 8, *B* and *C*), but (+)-(*S*)-bakuchiol only produced a weak inhibition of NA protein and A/CA/7/09 particle activity and did not inhibit A/PR/8/34 particle activity (Fig. 8, *A–C*).

**FIGURE 9.**
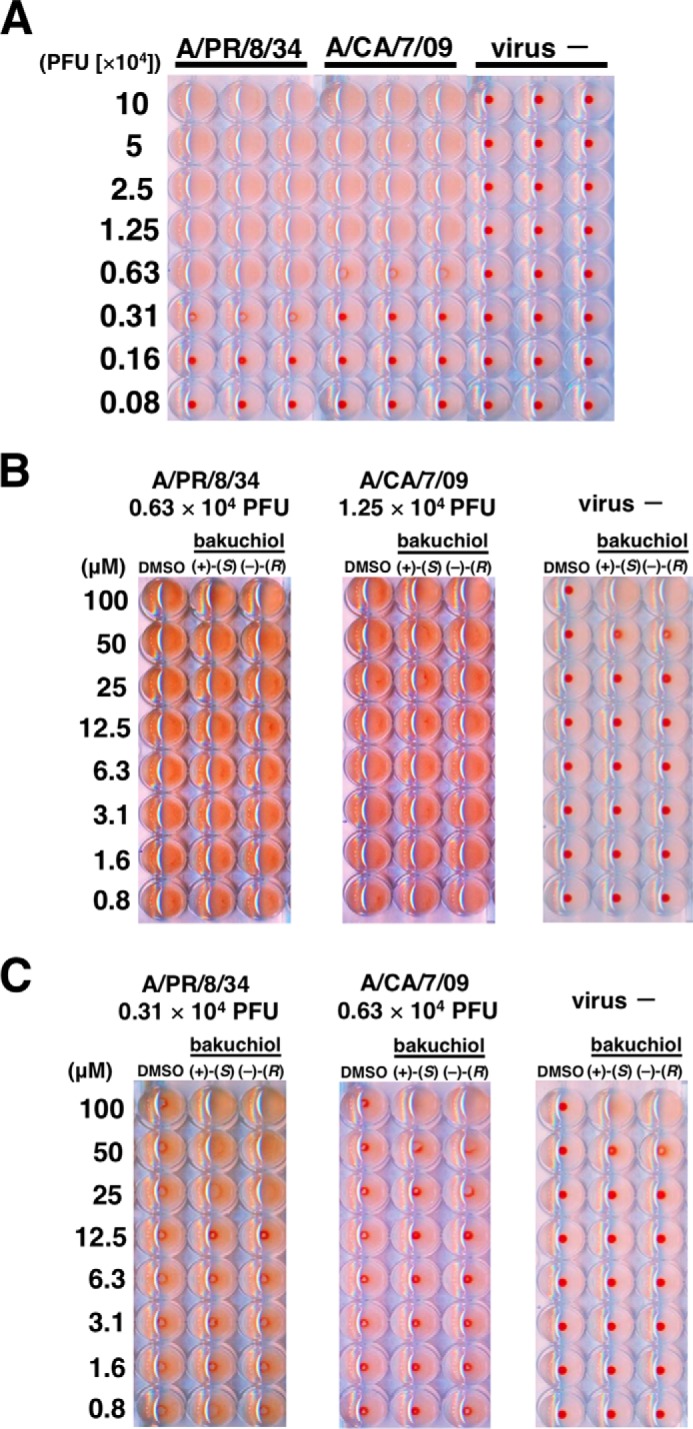
**Influenza A viral hemagglutination assay.** *A*, A/PR/8/34 and A/CA/7/09 were diluted with PBS to produce the indicated MOIs. Chicken red blood cells were added to each well, incubated for 1 h, and photographed. *B* and *C*, the indicated MOIs of A/PR/8/34 or A/CA/7/09 were added to the indicated concentrations of (+)-(*S*)-bakuchiol or (−)-(*R*)-bakuchiol, diluted in PBS, mixed with 0.5% chicken red blood cells, incubated for 1 h, and photographed. DMSO (0.008–1%) was used as the negative control. Data are representative of two independent experiments, and the results were reproducible.

**FIGURE 10.**
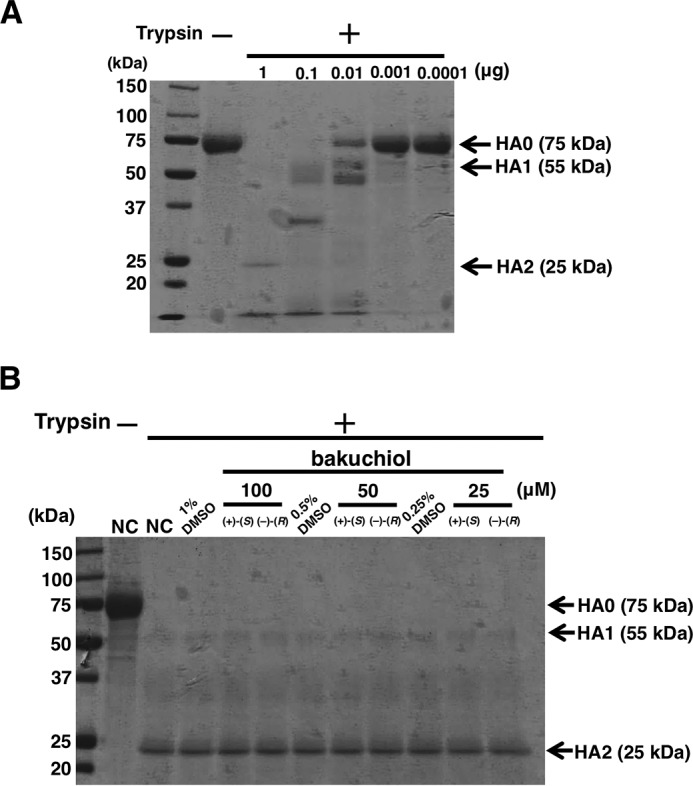
**Trypsin digestion of influenza A viral HA.** *A*, recombinant A/PR/8/34 HA protein was incubated in PBS (pH 5.0). The pH was adjusted to 7.5, the indicated amount of TPCK-trypsin was added, the mixture was incubated, and cleavage was visualized by SDS-PAGE and Coomassie Blue G-250 staining. *B*, recombinant A/PR/8/34 HA protein was incubated with the indicated concentrations of (+)-(*S*)-bakuchiol or (−)-(*R*)-bakuchiol at a pH of 5.0. The pH was adjusted to 7.5, 1 μg TPCK-trypsin was added, the mixture was incubated, and cleavage was visualized by SDS-PAGE and Coomassie Blue G-250 staining. Data are representative of two independent experiments, and the results were reproducible.

Next, we tested whether bakuchiol could inhibit the hemagglutination activity of A/PR/8/34 or A/CA/7/09 influenza viral strains. Chicken red blood cells were agglutinated by A/PR/8/34 or A/CA/7/09 (Fig. 9, *A–C*), and this activity was not inhibited by bakuchiol (Fig. 9, *B* and *C*). Influenza A viral HA0, the precursor of HA, is cleaved by cellular proteases like trypsin to produce HA1 and HA2, and this triggers the fusion of viral envelope and endosome membrane in an acidic environment (43–45). We therefore tested HA digestion by trypsin and could not detect any bakuchiol-mediated inhibition of this cleavage (Fig. 10, *A* and *B*).

**FIGURE 11.**
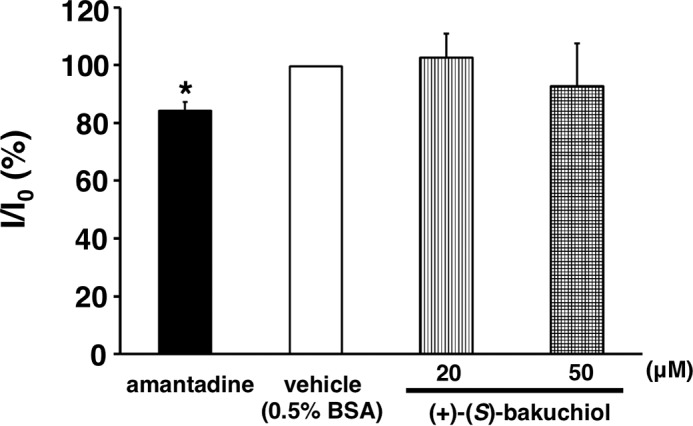
**Influenza A viral M2 channel assay.** Effects of (+)-(*S*)-bakuchiol (20 and 50 μm) and amantadine (100 μm) on the activity of M2 channels expressed in CHO cells, expressed as a percentage of the control cell amplitude (control) before drug application. Data represent the mean ± S.E. (*error bars*). *, *p* < 0.05 for the comparison with control.

We performed a patch clamp assay using Chinese hamster ovary cells expressing influenza virus A/PR/8/34 M2 in order to evaluate the effect of bakuchiol on this viral ion channel. Amantadine, the positive control, produced a weak inhibition of M2 ion channel activity, whereas (+)-(*S*)-bakuchiol did not inhibit this activity (Fig. 11). To explore why the activity of amantadine was weak, we sequenced the A/PR/8/34 M2 cDNA in the pCA-M2 plasmid and identified V27A and S31N mutations (data not shown). These mutations have been reported to produce amantadine-insensitive influenza A virus phenotypes (46–48). The M2 cDNA gene in pCA-M2 was cloned from the A/PR/8/34 strain used in the assays described above, where (+)-(*S*)-bakuchiol inhibited A/PR/8/34 infection and growth (Fig. 1, *lanes 3* and *4*) but did not inhibit A/PR/8/34 M2 ion channel activity (Fig. 11). Therefore, (+)-(*S*)-bakuchiol did not appear to target the A/PR/8/34 M2 ion channel. Taken together, these data showed that bakuchiol had no observable effects on the functions of influenza A viral surface proteins that were strong enough to explain its anti-influenza virus activity, suggesting that this compound may act on other targets within the influenza virus or the host cell.

##### Bakuchiol Induced Host Factor(s) That Inhibited Luciferase Activity

**FIGURE 12.**
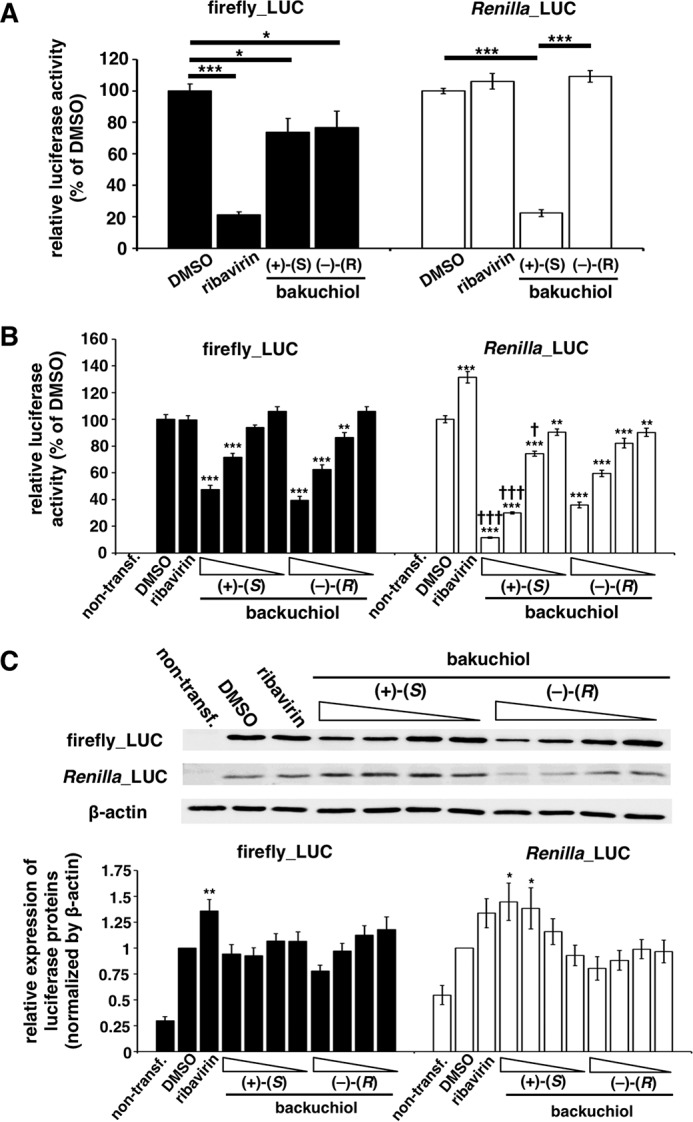
**Bakuchiol induced host factor(s) that inhibited firefly and *Renilla* luciferase activities.** *A*, a minigenome assay based on the Dual-Luciferase system was performed in MDCK cells. Firefly (Firefly_LUC) and *Renilla* (Renilla_LUC) luciferase activities were measured in lysates of transfected cells treated with the indicated compounds (*n* = 6): DMSO (0.5%), ribavirin (50 μm), (+)-(*S*)-bakuchiol (50 μm), or (−)-(*R*)-bakuchiol (50 μm). Each luciferase activity was expressed relative to that of the DMSO-treated cells (set as 100%). Data are presented as the mean ± S.E. (*error bars*) of two independent experiments. *, *p* < 0.05; ***, *p* < 0.001 for the indicated comparisons. *B* and *C*, MDCK cells were co-transfected with pGL3-control and pRL-TK-Rluc. The co-transfected cells were treated with (+)-(*S*)-bakuchiol or (−)-(*R*)-bakuchiol (1, 5, 25, or 50 μm), ribavirin (50 μm), or DMSO (0.5%, as a negative control) for 24 h. *B*, firefly and *Renilla* luciferase activities (non-transfection group, *n* = 7; other groups, *n* = 15) were measured by the Dual-Glo luciferase assay system. Each luciferase activity was expressed relative to the DMSO-treated cells (set as 100%). *C*, protein levels (*n* = 9) were evaluated by Western blotting. The level of protein was normalized to the β-actin level and expressed relative to the DMSO-treated cells (set as 1). Data are presented as the mean ± S.E. of 3–5 independent experiments. The results were reproducible. *One symbol*, *p* < 0.05; *two symbols*, *p* < 0.01; *three symbols*, *p* < 0.001.

Transcription and replication of the influenza A viral genome require the activity of a highly conserved RdRp (49). To evaluate whether bakuchiol inhibited influenza A viral RdRp, we used the minigenome assay employing firefly and *Renilla* luciferase reporters driven by the viral RdRp and the endogenous RNA polymerase II, respectively (32, 33). Ribavirin, a viral RdRp inhibitor (50), reduced firefly luciferase activity, as compared with the activity observed in the presence of DMSO, without affecting *Renilla* luciferase activity (Fig. 12*A*); this indicated that the assay detected ribavirin's selective inhibition of viral RdRp activity. As shown in Fig. 12*A*, (+)-(*S*)-bakuchiol and (−)-(*R*)-bakuchiol both reduced firefly luciferase activity, and (+)-(*S*)-bakuchiol also produced an unexpected reduction of the *Renilla* luciferase activity, as compared with that observed in the presence of DMSO. This finding suggested that (+)-(*S*)-bakuchiol inhibited influenza RdRp and also endogenous RNA polymerase II. We therefore transfected MDCK cells with plasmids expressing firefly and *Renilla* luciferases, without influenza RdRp. This study confirmed that (+)-(*S*)-bakuchiol and (−)-(*R*)-bakuchiol dose-dependently reduced these luciferase activities, as compared with DMSO (Fig. 12*B*), whereas ribavirin did not. (+)-(*S*)-Bakuchiol had a greater inhibitory effect on *Renilla* luciferase than did (−)-(*R*)-bakuchiol (Fig. 12*B*). Additionally, ≤50 μm (+)-(*S*)-bakuchiol and (−)-(*R*)-bakuchiol did not reduce the cell viability (Fig. 2). Therefore, these data confirmed that bakuchiol inhibited firefly and *Renilla* luciferase independent of RdRp, in the absence of any effects on cell viability. We considered three possible interpretations of these observations: (i) (+)-(*S*)-bakuchiol inhibited expression of the transfected luciferase gene in an RdRp-independent manner; (ii) (+)-(*S*)-bakuchiol inhibited the enzymatic activity of luciferase; or (iii) (+)-(*S*)-bakuchiol induced some host factors that inhibited luciferase expression or activity.

**FIGURE 13.**
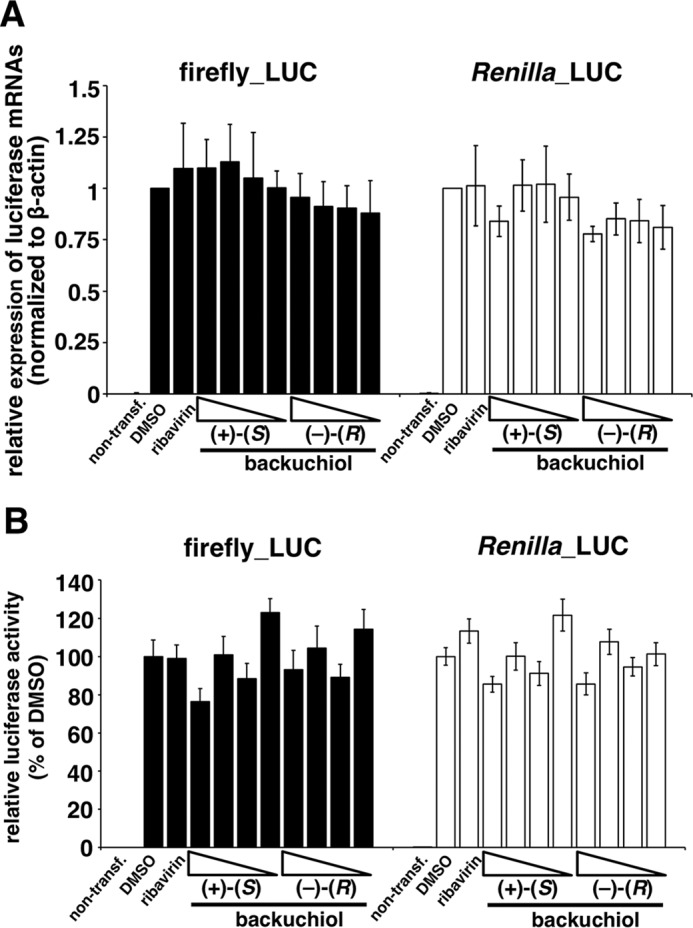
**Analysis of mRNA levels and direct inhibition of firefly and *Renilla* luciferases.** *A* and *B*, MDCK cells were co-transfected with pGL3-control and pRL-TK-Rluc. The co-transfected cells were treated with (+)-(*S*)-bakuchiol or (−)-(*R*)-bakuchiol (1, 5, 25, or 50 μm), ribavirin (50 μm), or DMSO (0.5%) as a negative control for 24 h. *A*, mRNA levels (*n* = 3) were determined by RT-qPCR. The level of mRNA was normalized to the β-actin level and expressed relative to the DMSO-treated cells (set as 1). Data are presented as the mean ± S.E. of 3–5 independent experiments. The results were reproducible. *One symbol*, *p* < 0.05; *two symbols*, *p* < 0.01; *three symbols*, *p* < 0.001. *B*, (+)-(*S*)-bakuchiol, (−)-(*R*)-bakuchiol (1, 5, 25, or 50 μm), ribavirin (50 μm), or DMSO (0.5%) was added to lysates of cells transfected with pGL3-control and pRL-TK-Rluc and incubated for 30 min before measurement of *Renilla* luciferase activity. Luciferase activity (*n* = 6) was expressed relative to that of the DMSO-treated cells (set as 100%). Data are presented as the mean ± S.E. (*error bars*) of two independent experiments.

To examine the first possibility, we analyzed the levels of firefly and *Renilla* luciferase mRNA (Fig. 13*A*) and protein (Fig. 12*C*). These were not reduced in firefly and *Renilla* luciferase-transfected MDCK cells treated with (+)-(*S*)-bakuchiol or (−)-(*R*)-bakuchiol, as compared with the levels observed in the presence of DMSO (Figs. 13*A* and 12*C*, respectively). This indicated that bakuchiol treatment did not affect the transfection efficiency.

To examine the second possibility, we analyzed whether bakuchiol directly inhibited firefly and *Renilla* luciferase activities *in vitro* (Fig. 13*B*). These enzyme activities were not affected by (+)-(*S*)-bakuchiol or (−)-(*R*)-bakuchiol (Fig. 13*B*). Taken together, these findings indicated that the third possibility, that bakuchiol induced host factor(s) that inhibited firefly and *Renilla* luciferase, warranted further investigation.

##### Bakuchiol Induced Nrf2 Activation and Up-regulated NQO1 and GSTA3 mRNA Levels

**FIGURE 14.**
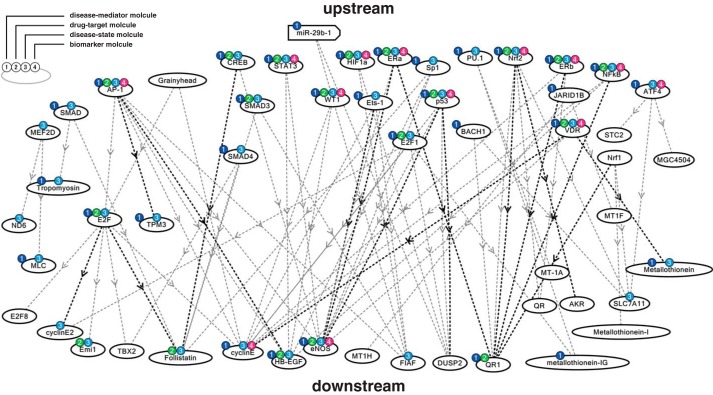
**Molecular network analysis by KeyMolnet using NGS results in bakuchiol-treated MDCK cells.** Using the NGS data, the molecular networks and pathways in bakuchiol-treated MDCK cells were analyzed by the KeyMolnet program *in silico.*

**FIGURE 15.**
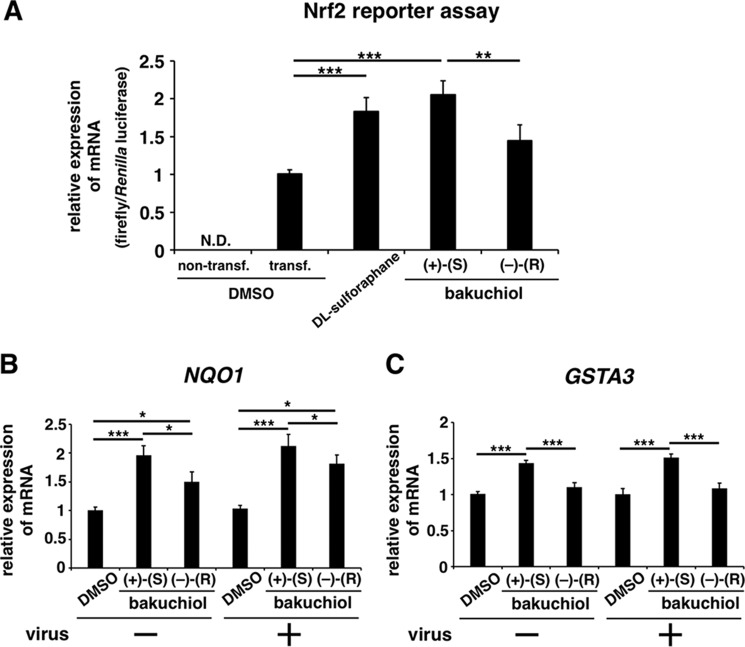
**Bakuchiol induced Nrf2 activation and increased the mRNA expression of Nrf2-activated genes in MDCK cells.** *A*, an Nrf2 reporter assay based on the Dual-Luciferase system was performed in MDCK cells. MDCK cells (1 × 10^5^) were transfected with pNQO1-ARE-luc, expressing a firefly luciferase gene driven by Nrf2 activation, and pRL-TK-Rluc, expressing *Renilla* luciferase driven by the herpes simplex viral thymidine kinase promoter. At 24 h post-transfection, the cells were treated with 25 μm (+)-(*S*)-bakuchiol (*n* = 9) or (−)-(*R*)-bakuchiol (*n* = 9) at 37 °C under 5% CO_2_. 0.25% DMSO (non-transfection (*n* = 9) and transfection (*n* = 9)) and 25 μm dl-sulforaphane (*n* = 9) were used as the negative and positive controls, respectively. The levels of firefly and *Renilla* luciferase mRNA were analyzed by RT-qPCR after 24 h and normalized to β-actin mRNA. The relative levels of firefly luciferase mRNA per *Renilla* luciferase mRNA were calculated and compared with that observed in the DMSO-treated cells (set as 1). *N.D.*, not detected; *transf*., transfection. *B* and *C*, (+)-(*S*)-bakuchiol (25 μm), (−)-(*R*)-bakuchiol (25 μm), or DMSO (0.25%; negative control) was mixed with A/PR/8/34 (MOI; 0.1) and added to 1 × 10^5^ MDCK cells for 24 h. Total RNA was extracted from cell lysates, and levels of *NQO1* (*n* = 8) (*A*) and *GSTA3* (*n* = 8) (*B*) mRNA were determined by RT-qPCR, normalized to β-actin mRNA, and expressed relative to the DMSO-treated non-infected cells (set as 1). Data are presented as the mean ± S.E. (*error bars*) of three independent experiments. *, *p* < 0.05; **, *p* < 0.01; ***, *p* < 0.001 for the indicated comparisons.

To investigate host factor(s) affected by bakuchiol, we performed NGS analysis of the MDCK transcriptome in cells treated with bakuchiol and influenza virus A/PR/8/34 (34) (supplemental Table 2). To identify molecular pathways activated by bakuchiol in the cells, we also performed molecular network analysis using KeyMolnet and the NGS results. This showed that bakuchiol activated the Nrf pathway (Table 2 and Fig. 14). We then analyzed whether bakuchiol activated Nrf2 using a Nrf2 reporter assay (Fig. 15*A*). This showed that (+)-(*S*)-bakuchiol and dl-sulforaphane, but not (−)-(*R*)-bakuchiol, induced Nrf2 activation (Fig. 15*A*). Furthermore, we found that mRNA levels of *NQO1* and GSTs, were up-regulated following exposure to (+)-(*S*)-bakuchiol or (−)-(*R*)-bakuchiol (Table 3). To confirm these findings, we performed a quantitative analysis of *NQO1* and *GSTA3* mRNA in MDCK cells treated with bakuchiol in the presence and absence of A/PR/8/34 using RT-qPCR. The levels of *NQO1* (Fig. 15*B*) and *GSTA3* (Fig. 15*C*) mRNAs in MDCK cells treated with (+)-(*S*)-bakuchiol or (−)-(*R*)-bakuchiol were significantly increased, as compared with DMSO-treated cells. (+)-(*S*)-Bakuchiol had a greater effect than did (−)-(*R*)-bakuchiol (Fig. 15, *B* and *C*), indicating a correlation with the enantiomer-specific anti-influenza virus activity of bakuchiol. It has been reported that the mRNA expression of *NQO1* and GSTs are regulated by the Nrf2 transcription factor and are related to the cellular response to oxidative stress (51–54). Taken together, these data demonstrated that bakuchiol activated the Nrf2 pathway.

**TABLE 2:** **KeyMolnet analysis of bakuchiol-induced expression**

Rank	Pathway	Score	Score (*p*)
1	Transcriptional regulation by Nrf	18.734	2.29E − 06
2	Estrogen signaling pathway	16.631	9.85E − 06
3	Transcriptional regulation by vitamin D receptor	9.714	1.19E − 03
4	Transcriptional regulation by AP-1	9.642	1.25E − 03
5	Transcriptional regulation by p53	7.877	4.25E − 03
6	Transcriptional regulation by RB/E2F	7.719	4.75E − 03

**TABLE 3:** **Analysis of mRNA expression in MDCK cells by next generation sequencing** The data indicate the number of reads per kilobase per million mapped reads. Also see supplemental Table 2. The entire data set has been deposited in the DNA Data Bank of Japan (accession number DRA003499) and in the Gene Expression Omnibus (accession number GSE73750).

Gene	Without A/PR/8/34			With A/PR/8/34		
	DMSO	(+)-(*S*)-Bakuchiol	(−)-(*R*)-Bakuchiol	DMSO	(+)-(*S*)-Bakuchiol	(−)-(*R*)-Bakuchiol
	*RPKM*			*RPKM*		
*NQO1* (NAD(P)H dehydrogenase, quinone 1)	113.4	202.7	159.9	74.5	192	157.9
*GSTA3* (glutathione *S*-transferase)	13	19.4	15.3	6.3	17.6	14.5

## Discussion

In the present study, we found that (+)-(*S*)-bakuchiol enhanced the survival of influenza A virus-infected MDCK cells and inhibited influenza A viral infection, growth, and gene expression; in addition, (+)-(*S*)-bakuchiol reduced the expression of influenza A virus-induced immune response genes in the host cells. We also found that (+)-(*S*)-bakuchiol induced the activation of Nrf2 and the up-regulation of *NQO1* and *GSTA3* mRNAs. This is the first report indicating that (+)-(*S*)-bakuchiol possesses anti-influenza virus activity. We found that the chirality of bakuchiol was important for this activity, and this should be considered when synthesizing bakuchiol derivatives as novel anti-influenza A virus H1N1 drugs.

We showed that (+)-(*S*)-bakuchiol had greater anti-influenza activity than (−)-(*R*)-bakuchiol, suggesting that the chirality of bakuchiol was important for this activity. Although the reason for this is still unclear, (−)-(*R*)-bakuchiol may have a reduced interaction with the target protein or be more easily degraded in cells, as compared with (+)-(*S*)-bakuchiol.

Our preincubation experiment showed that (+)-(*S*)-bakuchiol inhibited the H1N1 strains of the influenza virus (A/PR/8/34 and A/CA/7/09) but not the H3N2 strain (A/Aichi/2/68) (Fig. 1*B*, *lanes 3* and *4*). This may reflect strain differences in viral proteins or in the host cell response.

The HA and NA viral proteins differ between the H1N1 and H3N2 strains. It has also been reported that an anti-M2 ectodomain monoclonal antibody (clone rM2ss23) inhibited the viral replication of A/Aichi/2/68 and an A/PR/8/34 recombinant variant expressing A/Aichi/2/68-HA and/or M segment strains but did not inhibit the A/PR/8/34 strain (55). HA and M2 were co-localized in infected MDCK cells during virus budding (56), suggesting that strain-dependent differences in HA-M2 interactions might affect the inhibition of viral replication. Therefore, although bakuchiol did not inhibit the functions of A/PR/8/34 HA and M2 proteins (Figs. 9–11), it might affect their interaction while not affecting the A/Aichi/2/68 HA-M2 interactions.

Bakuchiol induced Nrf2 activation and up-regulated *NQO1* and *GSTA3* mRNA levels in MDCK cells (Fig. 15), indicating that it influenced the host response to oxidative stress. It has been reported that the host cell responses, including the innate immune response (57) and the cellular microRNA signature (58), differed following infection by H1N1 or H3N2 strains. Therefore, the different effects of bakuchiol on A/PR/8/34 and A/Aichi/2/68 strains may reflect differences in the MDCK host response to oxidative stress following infection with these viruses.

Nrf2 reporter assay, transcriptome, and RT-qPCR analyses in MDCK cells treated with bakuchiol and A/PR/8/34 showed that bakuchiol induced Nrf2 activation and up-regulated *NQO1* and *GSTA3* mRNA levels (Fig. 15). NQO1 catalyzes the reduction of various quinones via a two-electron mechanism involving NADH or NADPH, preventing the formation of free radicals and ROS. An increase in the level of ROS activates Nrf2 binding to the NQO1 promoter, increasing NQO1 production (59). Additionally, NQO1 stabilizes p53 in an NADH-dependent manner, promoting accumulation of p53 protein in cells (59). Chen *et al.* (15) reported that bakuchiol increased p53 expression and induced apoptosis via ROS-dependent reduction of mitochondrial membrane potential in A549 cells. Therefore, we speculate that the up-regulation of *NQO1* mRNA by bakuchiol is induced by ROS-dependent Nrf2 activation and increases the level of p53 protein in MDCK cells. Furthermore, Nrf2 up-regulation has been shown to reduce influenza A viral entry and replication (60), and the inhibition of p53 expression increases influenza A viral growth (61), suggesting that up-regulation of Nrf2 and p53 would inhibit influenza A viral growth. It has been reported that oltipraz (4-methyl-5(pyrazinyl-2)-1–2-dithiole-3-thione) and D3T (3*H*-1,2-dithiole-3thione), compounds that possess anti-cancer activities in multiple target organs (62), increase the Nrf2-driven expression of NQO1 (52, 63). Therefore, Nrf2 activation could represent one of the anti-influenza A virus H1N1 mechanisms of bakuchiol. However, because the direct target of bakuchiol remains unclear, further studies will be needed to explore this.

**FIGURE 16.**
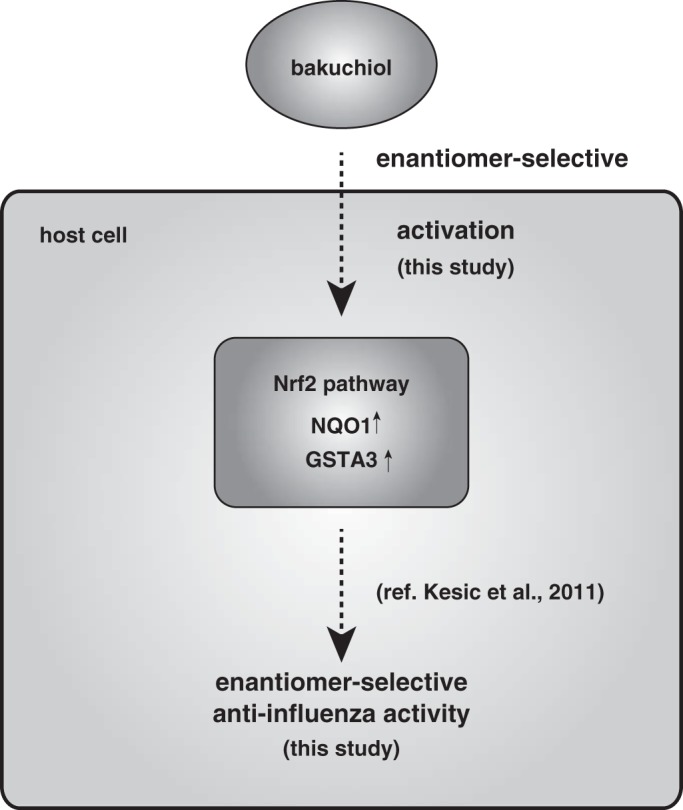
**Schematic showing novel mechanisms of anti-influenza virus activity of bakuchiol.** This scheme includes results and conclusions of the present study and previous report (60).

Based on the findings of this study and previous reports, as shown in Fig. 16, we suggest that anti-influenza virus activity by bakuchiol is involved in Nrf2 activation. In conclusion, the findings of the present study demonstrated that bakuchiol produced an enantiomer-selective anti-influenza A virus activity via a novel mechanism involving the host cell response. These data will contribute to the development of novel approaches to the treatment of influenza.

## Author Contributions

T. K. and M. S. designed the study and wrote the paper. T. E. and C. Y. synthesized and purified chemicals. M. S. and Y. A. performed anti-influenza virus assays. Y. S. performed next generation sequencing. S. Kohnomi and S. Konishi performed channel assays. E. T. and H. K. provided influenza viral strains. All authors reviewed the results and approved the final version of the manuscript.

## Supplementary Material

Supplemental Data

## Footnotes

2 The abbreviations used are:

NA: neuraminidase
MDCK: Madin-Darby canine kidney
Mx1: myxovirus-resistant protein 1
NGS: next generation sequencing
NQO1: NAD(P)H quinone oxidoreductase 1
Nrf2: nuclear factor erythroid 2-related factor 2
NP: nucleoprotein
MOI: multiplicity of infection
TPCK: l-(tosylamido-2-phenyl) ethyl chloromethyl ketone
NS1: nonstructural protein 1
rRNA: ribosomal RNA
CA: cytomegalovirus enhancer
RdRp: RNA-dependent RNA polymerase
RLU: relative light units
RPKM: reads per kilobase per million mapped reads
MTT: thiazolyl blue tetrazolium bromide
ME: marchantin E
RT-qPCR: reverse transcription quantitative polymerase chain reaction.
