# Supplementary material for: Bakuchiol Is a Phenolic Isoprenoid with Novel Enantiomer-selective Anti-influenza A Virus Activity Involving Nrf2 Activation
Source: J Biol Chem. 2015 Oct 7;290(46):28001–17. doi: 10.1074/jbc.M115.669465 (PMC4646038; doi:10.1074/jbc.M115.669465)
Supplement: Supplemental Data [file supp_290_46_28001__index.html]

Bakuchiol is a Phenolic Isoprenoid with Novel Enantiomer-Selective Anti-Influenza A Virus Activity Involving Nrf2 Activation — Bakuchiol Is a Phenolic Isoprenoid with Novel Enantiomer-selective Anti-influenza A Virus Activity Involving Nrf2 Activation — Enantiomer-selective Anti-influenza Activity of Bakuchiol — Supplemental Data 

# Bakuchiol Is a Phenolic Isoprenoid with Novel Enantiomer-selective Anti-influenza A Virus Activity Involving Nrf2 Activation

## Supplemental Data

- Supplemental Table 1 (.xlsx, 11 KB) - Quantitative real-time PCR primer sequences.
- Supplemental Table 2 (.xlsx, 23 KB) - The gene list of > 1.5 fold-changed mRNA expression in (+)-(S)-bakuchiol-treated MDCK cells by the analysis of next generation sequencing.
